# Initiation to street life: a qualitative examination of the physical, social, and psychological practices in becoming an accepted member of the street youth community in Western Kenya

**DOI:** 10.1186/s12889-015-1942-8

**Published:** 2015-06-20

**Authors:** Juddy Wachira, Allan Kamanda, Lonnie Embleton, Violet Naanyu, Susanna Winston, David Ayuku, Paula Braitstein

**Affiliations:** Academic Model Providing Access to Healthcare (AMPATH), P.O. Box 4606, 30100 Eldoret, Kenya; Moi University, College of Health Sciences, School of Medicine, Eldoret, Kenya; Moi Teaching and Referral Hospital, Eldoret, Kenya; Brown University, Warren Alpert Medical School, Providence, RI USA; Rhode Island Hospital/Hasbro Children’s Hospital, Providence, RI USA; Department of Medicine, Indiana University, School of Medicine, Indianapolis, USA; University of Toronto, Dalla Lana School of Public Health, Ontario, Canada; Regenstrief Institute, Inc., Indianapolis, USA

**Keywords:** Initiation practices, Street connected children and youth, Gender, Sexual practices, Kenya

## Abstract

**Background:**

The objective of this study was to describe the physical, social, and psychological initiation practices of street connected children and youths, in Eldoret, western Kenya.

**Methods:**

This qualitative study was conducted from August 2013 to February 2014. A total of 65 SCCY aged 11–24 years were purposively sampled from the three referral points: 1) A dedicated study clinic for vulnerable children and youth at Moi Teaching and Referral Hospital (MTRH); 2) Primary locations in which street children reside “bases/barracks”; 3) Street youth community-based organizations. In-depth interviews and focus group discussions were used to collect data. All data were audio recorded, transcribed, translated to English, and a content analysis performed.

**Results:**

The overall median age was 18 years (IQR 14–20.5 years) and 69.2 % of participants were male. None had gone beyond primary level of education. The majority (81.5 %) reported to be sexually active. The street community had well-defined structures and rules that were protective of members and ensured survival on the streets. To be fully accepted children had to go through an initiation ritual that had important gender differences. Common rituals between males and females included interrogation, smearing of black soot, and payment of tax. Ritual practices unique to boys were physical abuse, theft of personal possessions, volatile substance use, being forced to eat garbage, and sodomy among the physically weak. Rituals unique to girls were being forced to ‘become a wife or sexual partner’, rape, and gang rape. Physical and psychological abuse during initiation was normalized and there were no clear mechanisms of dealing with these forms of abuse.

**Conclusion:**

There were important gender differences in the initiation practices of SCCY. Normalization of physical and psychological abuse during initiation contributes to the high health risks faced by these SCCY. Appropriate interventions need to be developed in collaboration with SCCY.

## Background

Worldwide, it is estimated that tens of millions of children live on the street, and the vast majority of them are from low- and middle-income countries [1]. In Kenya, it is estimated that 2.6 million children are orphaned [2], some of whom end up on the streets due to situational factors that arise from the loss of their parent(s). In 2007, it was reported that between 250,000-300,000 children lived and worked on streets across Kenya [3]. Effective approaches to address this concern are needed in order to mitigate the negative consequences of children living on the streets.

Generally across low- and middle-income countries including Kenya, situational factors such as poverty, political instability, violence, physical/sexual abuse, being orphaned, drug abuse, unplanned pregnancies, and family conflicts [4–8] have been reported to predispose these children to street life. Once on the streets, children form a strong social network with well-defined leadership roles, group dynamics and rules [8–10]. The majority of these street children are illiterate [11, 12] and have limited or no contact with their families [7, 12]. Hence, they rely on their street networks to provide them with the physical, psychological, and social support they would have otherwise received from their families [7, 8, 12]. Unfortunately, these social networks as well as the hostile street environment, promote early sexual practices [13–16], physical abuse, and drug and alcohol use [16–18], as coping mechanisms. This makes street connected children and youth (SCCY) a highly vulnerable group.

Among SCCY, group dynamics are complex because they differ by gender and cultural setting [8]. The street environment tends to favor boys who are more dominant and aggressive, compared to girls who are perceived as sexual beings [8]. Children needing acceptance into the street community have to conform to set street rules and regulations, which may have detrimental outcomes on their overall wellbeing [9]. Health outcomes such as mental illness, injuries, and infectious diseases such as STIs and HIV are therefore common in this community [16, 19, 20]. Therefore understanding the role that initiation rites play in the wellbeing of SCCY is critical to defining appropriate interventions for this vulnerable group.

Generally, health outcomes among SCCY have not been well described [16, 21]. In our effort to understand the sexual behaviors of SCCY we uncovered the importance of initiation processes for boys and girls into the street community and that they played a key role in their overall wellbeing and socialization into street culture. There are very few studies in Kenya that have sought to understand what SCCY go through before they are fully accepted into the street community [8, 18]. Understanding these ‘rites of passage’ will inform the development of programs that can be responsive and appropriate to the lives of SCCY, whether through harm reduction or reintegration into the society. Furthermore, it will provide organizations working with street children insight into some of the behavioral and health issues common amongst this vulnerable group, as they design appropriate interventions for them. The objective of this study was to describe the physical, social, and psychological initiation practices of street connected children and youths, in Eldoret, western Kenya.

## Methods

### Study design

This qualitative study was conducted from August 2013 to February 2014. The overall goal of the study was to explore the sexual language and practices of SCCY in western Kenya. Initiation practices emerged as a key finding which informed the analysis and the focus of this paper. In-depth interviews and focus group discussions (FGD) were used to collect data.

### Study setting

Uasin Gishu (UG) County is one of the 47 counties in western Kenya. In 2010, UG County had approximately 894,179 individuals, of whom 41.5 % were aged 14 years or less [22]. Approximately, 51.3 % of the County population live below the Kenyan poverty line [23]. The headquarters of this County is Eldoret town, which has a population of 289,389 [23].

### Study population

We targeted “full-time” and “part-time” SCCY aged 11–24 years who had lived on the street for more than 3 months. We considered “full-time” SCCY as those who lived on the streets on a full time basis or shared a shelter at night with other SCCY. While “part-time” SCCY were those who lived on the streets during the day and at night went home to an adult parent(s), relative or guardian.

### Human subjects protection

Ethical approval was obtained from the Moi Teaching and Referral Hospital (MTRH) Institutional Research and Ethics Committee (IREC) as well as the Indiana University Institutional Review Board (IRB). All interview sessions were conducted in private rooms. Prior to the interviews, trained research assistants provided SCCY with verbal information about the study and assessed their willingness to participate. Written consent was obtained from willing participants aged 18 years and older. Written assent was required for those aged 11–17 years, in addition to written guardian consent from the UG District children’s office. Fingerprints were used for children who were unable to sign or write their names. A child psychologist was present during all interviews sessions for children aged 11–17 years, in order to provide counseling in the event of psychological distress during the sessions. Privacy and confidentially were assured at all times. Participants were requested to talk about their general perceptions and observations about sexual activities in the street community and not about their own personal experiences. In addition, participants were asked not to disclose their full names and/or those of other SCCY. First names of participants were used to facilitate discussions during FGD sessions.

### Sampling and recruitment

Study participants (SCCY 11–24 years) were purposively sampled from three points: 1) A dedicated study clinic for vulnerable children and youth at MTRH; 2) Street venues “bases/barracks” which are the primary locations that SCCY reside; 3) Street youth community-based organizations. Extensive street outreach and study sensitization occurred at these sites to establish rapport and trust with SCCY. A pre-existing relationship between the research team and a number of SCCY in Eldoret assisted in identification and outreach within these locations. A street outreach worker with experience working with this population was engaged to conduct the outreach and provide information about the study. Once the purpose of the study was explained, SCCY were invited to participate voluntarily in the investigation. Convenience sampling was used to recruit participants.

### Study instrument

A series of 10–15 open-ended questions previously pre-tested among SCCY in UG guided the individual interviews and FGDs. The main interview domains included: 1) Initiation on the streets; 2) Types of relationships established on the streets; 3) Sexual practices (acceptable and unacceptable) and behaviors; 4) Language used for sexual acts; 5) Reproductive health; 6) Sexually transmitted infections and health; 7) Sexual abuse and rape; 8) Roles and responsibilities of leaders on the streets. In addition, a set of structured questions on basic socio-demographic information including age, gender, educational level and area of residence were incorporated. An additional domain was incorporated for interviews with barracks leaders, to explore their specific roles in the street community. The guides for the younger participants (11–13 years) were modified in order to reflect developmentally appropriate questions regarding sexual activities.

### Procedure

We conducted a total of 25 in-depth interviews and 5 FGDs (8–12 participants) with SCCY in the following age groups: 11-13 years, 14-17 years and 18-24 years. Table 1 shows the distribution of interview sessions per group. We were unable to conduct FGDs with females aged 11–13 years. This was due to the low numbers of this group living on the streets that made it difficult to recruit them into the study. For both in-depth interviews and FGDs, eligible participants were referred to the study clinic at MTRH where all interview sessions were conducted in private rooms. This location was chosen to provide a neutral, safe, and private environment where SCCY could feel more comfortable discussing their sexual and reproductive health practices. Once written consent or assent was obtained, participants were first invited to take part in the interviews. The FGDs preceded the in-depth interviews to allow for further exploration of issues that may have emerged in the FGDs. Hence, participants for the in-depth interviews were identified following the FGDs. At the end of the FGD sessions, the study team invited willing participants to engage in the in-depth interviews, schedule on a later date that was convenient for the SCCY. The individual interviews took an average of 40 min while the FGDs took an average of one and half hours. All sessions were audio-recorded and conducted by two of the study investigators in Swahili, the Kenyan national language. For the FGDs, a trained research assistant was present to take notes. Transport reimbursement of Kenya Shillings 100 (~USD = 1.15) was provided after the interview sessions. Given the cost that participants incurred in getting to the study clinic, this amount was considered adequate and not enough to coerce or influence participation in the study.

**Table 1:** Distribution of interview sessions

Category	In-depth Interview sessions	FGDs sessions
Barrack leaders	4	0
Male		
11-13 years	1	1
14-17 years	6	1
18-24 years	7	1
Female		
11-13 years	0	0
14-17 years	3	1
18-24 years	4	1
Total	25	5

### Data analyses

Recorded interviews were transcribed and translated to English. The data were then coded and themes related to initiation practices were identified. Concepts from different interviews were then pooled together and integrated into common themes. Concepts from these themes were generated and used to organize the presentation of the results. For validation, independent coding and identification of themes were conducted by four investigators. The final write up consisted of summaries, interpretations and textual excerpts.

## Results

A total of 65 SCCY participated in the study. One 14 year old boy declined to participate in the study while two participants (one female and one male, both 18 years old), could not participant in the study because they were intoxicated. As shown in Table 2, the median age was 18 years (IQR 14–20.5 years) and 69.2 % were male. None had gone beyond primary education and only 24 % were part-time SCCY. The majority (81.5 %) reported being sexually active.

**Table 2:** Characteristics of street youth

Variables	N (%)/ M(IQR)
Age in years	18 (14–20.5)
Gender	
Male	45 (69.2 %)
Female	20 (30.8 %)
Education level	
Nursery and none	14 (21.5 %)
Lower primary school (Std1-4)	23 (35.4 %)
Upper primary school (Std 5–8)	28 (43.1 %)
Type of street child	
Spend all day and nights in the street	16 (24.6 %)
Spend days in the street and nights home with other street children	33 (50.8)
Spend days in the street and nights home to an adult	16 (24 %)
Currently sexually active	
No	11 (16.9 %)
Yes	53 (81.5 %)

### Street community structure and leadership

‘Mshefa’ is a Swahili slang word that means a hustler (one who works hard to survive). This is a common term used by the street community to refer to a street member, either male or female.

The street community is divided into sub-groups that have well established territories within defined geographical areas known as, *bases or barracks*. During the study, we identified seven major barracks in Eldoret namely California, Juma Haji, Asis, Barrack ya Stevo, Mangula, Jua Kali, and Eastleigh. Barracks members are restricted to engage in street activities such as theft or selling of scrap metals within the set boundaries of their barracks. Failure to respect boundaries of other groups results in physical battles between the members of different barracks. Clear rules and regulation that govern each barracks are therefore established to avoid conflict.

Each barracks has a leader known as a barracks/base leader. The barracks leader has the responsibility of protecting members, especially the young ones, from any form of attack whether physical or psychological including verbal and sexual abuse. They also organize members to provide financial support in the event of a medical emergency or the death of a member Barracks leaders ensure discipline is maintained and have the mandate of deciding the kind of punishment a member faces, if they go against the stipulated rules and regulations. They also play a key role in the initiation of new SCCY boys and girls into their community. They ensure that the rituals are upheld and are normally consulted in the event of any arising issue regarding the induction of new members into street community.

Leadership is either attained by force or democratically, hence a majority of the leadership roles were taken by males. Using force entails establishing a group of followers or a gang, who engage in physical battle with supporters of the ruling leader. Whichever group wins the battle is accepted as the ruling gang and the leader takes over the barracks’ leadership role.

**Barracks leader (22 years):***I overpowered them with my group. I also had my own group called black matches. So we went at their base then ambushed them and overpowered them. They came under us as I became the leader. Since then when anybody has a problem they call me.*

The democratic process follows the standard voting system and mainly occurs when a leader dies or members are disgruntled with the leadership in place. Candidates are either nominated or nominate themselves.

**Barracks leader (22 years):***If there is someone not satisfied with your leadership, they can complain and fresh elections will be done....but it also depends on how you relate with others. If you have a bad relationship with the people, you won’t be elected. We are judged by how we treat others, especially the young ones…we can be like five candidates. So the street members will queue and elect. There is a small piece of paper and each member will specify the candidate they prefer. The name of the candidate will be written on the piece of paper, folded and cast in a bucket. And after that the votes are counted.*

### Initiation to the street community

**Fig. 1:**
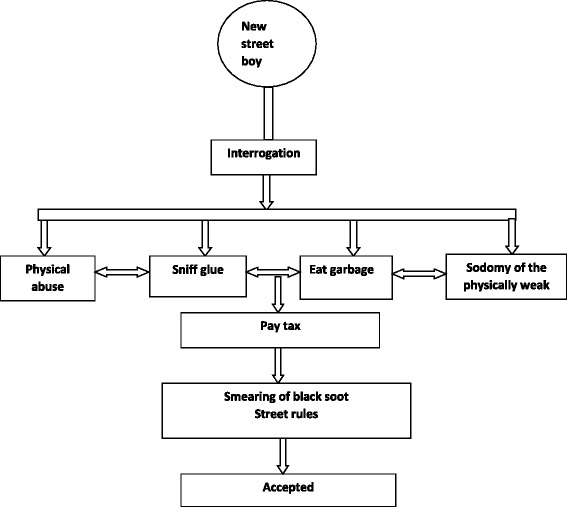
Initiation process of SCCY boys

**Fig. 2:**
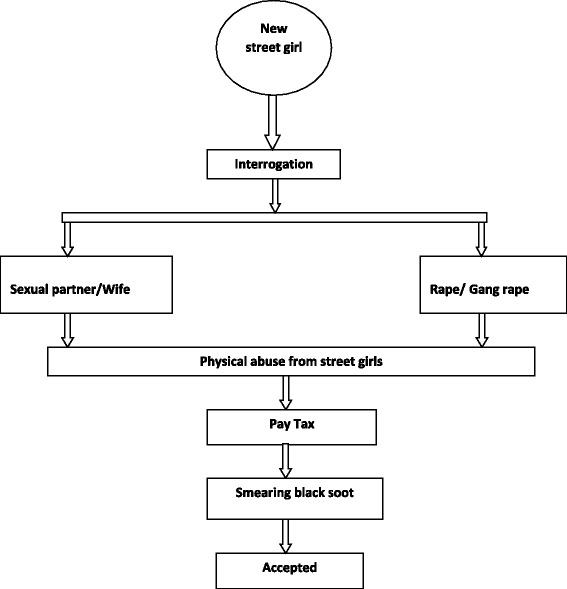
Initiation process of SCCY girls

There were unique differences in the way boys and girls were inducted into the street community. However, there were also some common initiation practices. Figures 1 and 2 shows the initiation practices for both street boys and girls, respectively.

The first initiation step involves an interrogation session by the barracks leaders. This is done to determine the origin of the SCCY and the reason for relocation to the streets. Those who provide valid reasons (orphaned or unbearable home environment) are easily accepted, however those with no justifiable reason are forced back to their homes.

**Male FGD (14–17 years):***If he comes and let’s say he is new in a group, they ask him questions.... What made you leave home? He can tell you the problems that made him leave his home.... So after telling the others what made him leave his home, they understand that truly this one had problem.*

**Male (22 years)***: I tell the other members of the barracks if any child comes to the street, he/she should be asked where they come from and if it is near the town, they should be beaten so that they go back home. They should not be accepted to stay in town*.

Once the street boy or girl has been deemed eligible to join the group, other rituals are performed at no particular order. Paying tax was considered essential. It was stated that a new SCCY had to pay money ‘tax’ to the older street members. The amount of money varied from Kenya Shillings 5 to 50 per day over a period of 3 to 7 days. Failure to pay tax would result to physical abuse and for the girls, gang rape.

**Male FGD (18–24 years):***They ask you how much money you have, and don’t try to lie to them. Tell them you have a certain amount of money and you would like them to share it with them as brothers. But if you lie to them, they will beat you, take the money or your shoes and good clothes from you.*

**Male FGD (11–13 years)**: *I came from Langas (a large peri-urban settlement) and I met a boy who took me to Juma Haji (SCCY barracks) to sleep there, and I had to ‘rwatisha madimbo’ (collect scrap metals). I gave him the money and he introduced me to some of the boys there. After a while we were chased away and we went to Langas where we got chased again and came back to California (SCCY barracks)....They told me to give them money first before I could become one of them****…****Kenya Shillings 5 or 10 or 20, but the big ones ask for Kenya Shillings 50.*

**Female (21 years):***If you don’t have money ‘msoto’ (means being broke), they will not like you. They want somebody who they can borrow money when they don’t have. So if they ask for money and you say you don’t have, they can even send a group of ten boys to have sex with you. We usually call it “combination.” So when they do that they think it might make you go back from where you came from.*

Another defining induction process common to girls and boys was smearing of black soot (from burnt tires) over the entire body of a new SCCY. This was considered as a form of oath.

**Male (19 years):***They smear you with ‘bamba nyeusi’ soot from a burnt tire. They smear you with that and that now makes you part of them… After smearing you with the ashes, you can go to the river and wash or just walk around… It is like taking an oath so that you become a member of that base, so that they know you are one of them*

### Initiation practices unique to boys

Physical abuse formed a great part of the initiation process for the boys. Physical abuse was not only a way of assessing the physical strength of the new street boy but also a way of evaluating his level of resilience to survive on the street.

**Male FGD (18–24 years):***Like for my case when I came I went through that; I was beaten and all the money I had was taken away from me.*

**Male FGD (14–17 years):***If you have more strength than others… you fight to show you have strength.*…y*ou beat others… Yes, and then they fear you.*

As the new street boys were physically abused, their personal possessions were also taken away and shared among the older SCCY. The older street boys took the items that they considered expensive and either sold or personalized them. This was a way in which the older boys showed dominancy over the new members.

**Female (17 years):***If it is a new boy and he came with some things like a phone or money, they will snatch them from him, smear the boy with the ash from burnt tires and beat him telling him that he came to take their wives. There is a difference in the way boys and girls are welcomed at the base....I don’t know but it could be because they are used to doing such things in their life because most of them are thieves. You know it is not all of them who stay on the streets. Some street boys have rented houses and they believe that when they snatch such things, they will sell and get some money to provide for their wives.*

Although sodomy was not condoned in the street community, instances were reported of new street boys, identified as physically weak, being sodomized. This meant that the majority of those sodomized were younger boys. It was reported that establishing a good rapport with the base leader was critical to avoiding any form of sexual abuse.

**Male FGD (18–24 years):***Madam, I want to tell you there are those who take the new boys by force and rape them on their behinds. They cheat them to take them to Kipchoge (a stadium in Eldoret). Like there is a man who passed away, he was called Copral; he could catch you, beat you up, take away your money, take you to a forest and rape you on your behind. Even if it is a boy.*

**Female (21 years):***But sometimes they also do it in the anus. For those who join as new members on the streets, they sometimes force them to engage in anal sex regardless if it is a boy or a girl.*

**Male Barracks leader (22 years)**: *On the side of boys, you know boys, even our boys here are not very good. You will find a big boy doing very bad things to a small boy. The guy will do anal sex with the child. They will do that to the child because they know the child is new and since they usually do that, he will buy the child some food then tell the child let’s go this way or send the child severally and the last time he will tell the child to bend and he does it to the child.*

Sniffing glue also formed part of the initiation process among boys. New boys were immediately inducted into this habit as a ways of promoting unity among street members.

**Male (17 years):***They expected me to react like them, to do what they are doing… There is sniffing glue, smoking (glue); you know there is unity in every base.*

In addition, new street boys were also forced to eat food from garbage bins/sites as a way of determining their readiness to survive in the harsh street environment.

**Male (24 years):***You can even sniff for a whole month. Then, you also have to eat the foods that they eat there on the streets and you know it is usually food remains. Sometimes food mixed with dirt but if you don’t eat the food, then you will not be taken as a member*.

New street boys were also informed of the barracks rules and regulations. However, there were times when the information provided varied depending on the intention of the older street boy giving out the rules. Older street boys were reported to take advantage of the naivety of the new street boys.

**Male (17 years):***Like you can’t mess up with one of the base members and then you go free. So they tell you as a base member that you should not see your fellow base member being beaten and then you don’t help them. And if you get something like food you share with each other, but if you get money that is just yours.....You know there are those people who behave badly and others well, every person with their behaviors. And there are those who can give you bad advice, they tell you to go and steal and another can tell you, you do that to get money. So those are the advice you get there, everyone gives you advice according to what they think.*

### Initiation practices unique to girls

Generally, the street community viewed girls as sexual beings, hence initiation and survival on the streets was associated with engaging in sexual acts, either willingly or by force. The sexual acts included vaginal, anal, and oral sex. Street girls believed that the initiation of girls was more brutal compared to boys. **Female FGD (14–17 years)**: *Madam Girls suffer more than boys* (referring to the initiation practices). Girls are referred to as ‘mboga ya jeshi’ meaning vegetables for the street soldier (older street boys). This statement denotes that girls are sexual objects that are available to any street boy. New street girls were therefore in high demand because they were viewed as ‘fresh vegetables’ for the street soldiers.

Similar to boys, new girls were interrogated before induction. However, the interrogation formed part of a seduction process with older street boys hoping to gain a new sexual partner or wife. The girls tended to receive a deceptively warm reception from the street boys who hoped to establish a sexual relationship with them. These sexual advances were not necessarily limited to one suitor.

**Male (20 years):***Let us say like me now, I do not have a girlfriend and my wife ran away. If a new girl comes I will sit down with her and have a talk and try to advise her. I will ask her where she is from, ask her if she did something wrong at home whether she used to go to school and then ask her if she would like to get married. If she refuses I tell her that there is a certain Home that helps people if you want to go back home you talk to a teacher there who will take her back home. If she accepts to get married. I take her.*

**Male FGD (11–13 years):** W*hen a girl comes to the base, they receive a warm reception; one boy takes her to his house and makes her his wife…*

**Male (20 years):***When a girl comes to the streets you get together and start having sex…..Anyone has sex with her… She can be with me today and be with someone else tomorrow. She will get used to it and after a while she will be like a member.*

Unfortunately, if the new girl refused sexual advances from the street boys, they place themselves at a higher risk of being gang rape. Older street girls therefore encouraged the new girl to form a sexual relationship with one of the street boys in order to avoid being gang raped. Having a sexual relationship with a street boy ensured that they remained protected from any aggressive group sexual advances or torture.

**Female (20 years):***The boys at the base must also welcome the girl by raping her although if she agrees with one of them, he can take her as the wife and then he will be the one to protect her.*

**Female FGD (14–17 years): ‘***wanakupiga kombi’ (gang rape) and when you accept you are accepted… They do a combination on you (gang rape)… they make a queue for you…the base members, the very big street boys*.

**Male FGD (18–24 years):***When you are new and you are a girl, maybe you have been brought by a girl who is your friend to the base, so when you come to the base there is a boy who will see you and he will tell his friends about you and how beautiful you are and they will call each other. They then ‘collaborate' (gang rape). If she is a virgin they rape her and leave her.... If she is a virgin you break her virginity....going after her in a large group, making a queue for her; when one is through another one goes in, all of them.*

Older street girls also sometimes benefited from the sexual encounters between the street boys and new street girls.

**Female (21 years):***She can be told by the older street girls to go to the older boys so that they do love to her then they take the money. So the girl will be used but it will be other girls that she walks with are the ones who will benefit by taking money from the boys. They will receive the money from the boys for the service of taking to them the new girl.*

In addition, the new girls also faced physical abuse from the older street girls as a way of expressing dominancy.

**Female (17 years):***Sometimes you will find the girls are also harassing a fellow girl, telling her that she came to take their husbands, beat her and even cut her with broken bottles. So if the girl is a person who gives up quickly she will decide to go but if she can stand it, she will just plead with the other girls then join the base and survive with them.*

### Dealing with rape

Given that rape formed a significant part of the initiation process we further explored what actions were taken when a street boy or girl was raped. Sodomy was viewed as graver offence than gang rape among girls. Street boys who were reported to have sodomized another street boy were punished or even chased out of the barracks.

**Male (20 years)**: *eeeeeheeh! we beat him up! We beat him up like a dog inside a mosque. After that he will be a changed person if he wants and if he continues we tell him we will take a hot coal and put it on his ‘thing’ (penis). So that he understands the rules we burn it (penis) just a little bit. And we tell him that is now like a medicine that will help him rectify his behavior.*

**Male (22 years):***When you do that (sodomy) you will receive a beating. We take you to “Pilato” (Pilate like in the bible) that is “Mahindra” (barracks leader). You will be beaten then expelled from the base. Such expelled people are the ones you will get in “Eastleigh and West” you cannot get them in town. Even the ones who do anal sex with children “mamende” (homosexuals) we beat them and expel them too.*

In addition, there was mention of a well-wisher in the larger community who SCCY felt they could run to in the event of sodomy. Even though this well-wisher did not take any legal action the SCCY expressed a sense of trust that perpetrators would be punished by this individual.

**Male FGD (14-17 years):***There is a man called ‘Mzee John’ he sells scrap metals, he is always helpful to us ‘Mashefa’.... He will go and beat them up.*

Unfortunately, for girls, the majority reported that no action was taken when girls were raped or gang raped by fellow street boys, because it was somehow acceptable. In fact there were instances when money was exchanged between the street youth perpetrator and the barracks leader to cover up the act. Sadly, reporting to the authorities was futile because the police believed that if the perpetuator was a street youth, then the street community should deal with it.

**Female (21 years):***Yes rape is acceptable. If you are being forced to have sex or being raped by a fellow street boy and another person passes there and sees the action, he will just tell you to cooperate and finish but will not interfere. Even the leaders of the base cannot help. He will just tell the boy to give him something small so that he doesn’t call a group of boys to beat him.*

**Female (17 years)**: *If you go at the police station there is nobody who will help you because they usually chase us away. They usually say that street people rape each other and they don’t even want them to appear at the station*.

**Male FGD (18–24 years)**: *You know “Gavaa” is just a name for a police, if you are “mshefa”(street youth) and you go to the police, they just say that you know each other …You know each other…You know streets is just considered as one thing…One thing, so if you go to the police they say you know each other, aha another one?…If you rape a girl even if she is not from the base, when she goes to report, the first thing they will ask is “where have you been raped”? And if it “mangula” (SCCY barracks) they ask you if you know the person who has raped you, they also tell you that, that place is dangerous, “Why did you choose to pass through there?” so it depends on where you have been raped. So if you have been raped at the base…There is no problem; there is nothing they can do. You can’t be caught.*

The only time when action was taken by the street community was when the perpetrator was not a street boy or when the victim was married or in a sexual relationship with a street boy.

**Female (21 years):***If a person who is not a member of the street rapes a girl, all the street people will look for him and beat him. They say that the person is coming to spoil their vegetables (street girls).*

Surprisingly older street girls did not see the need to protect new street girls from rape. They believed that because they went through the same ordeal, the new street girl should also experiences the same and viewed rape as an act of hardening the new girl. They therefore supported the act even if it was committed by their own sexual partners or husbands.

**Female FGD (14–17 years):***Yes, because the same was done to me. It hardens her. I will accept it because I want her to feel what I felt…okay, aha.****[Interviewer: So you will allow your man to do a ‘combination’ (gang rape) on another girl because the same was done to you?]****…I will accept.*

## Discussion

Our findings reveal the harsh reality that SCCY face before being fully accepted into the street community in Eldoret. There is a clear mechanism for street induction, survival, and protection of members that excludes children who are viewed as not belonging on the streets. Gender differences during induction were reported and are extremely important in understanding the risks that incoming SCCY are exposed to. Boys mainly experienced physical abuse and humiliation to prove their worth and resilience. Girls were sexually abused and like other studies have shown [15], they were viewed primarily as sexual objects. Rape and gang rape was the most common and accepted initiation ritual for girls. Though not uncommon, sodomy among new street boys was experienced by the physically weak; however unlike those that rape girls, perpetrators of sodomy were severely punished by street members. It is possible that the normalization of sexual abuse especially among girls and physical abuse among boys has promoted a cycle of abuse and violence on the streets. This contributes to the adverse health outcomes [14–16, 19] reported in this community. Our data provide important insights on the physical and psychological risks faced by street children that policy makers and program implementers could utilize to develop appropriate interventions.

Similar to gang initiation practices [24–26], incoming SCCY are forced to engage in deviant activities as a test of commitment to the street community. Generally, girls believed that their induction was more traumatizing than boys. The fact that street girls were devalued [15] and viewed as ‘vegetables’ for consumption by all street boys, highlight the low level of self-efficacy instilled in these girls. It was therefore not surprising that the older street girls, who had been socialized to believe this about themselves, bullied the new street girls and even promoted gang rape. On the other hand, boys are generally physically dominant, hence street girls may not have the physical ability to stand up against their fellow street boys and protect incoming street girls. The acceptance of rape or gang rape as a form of initiation ritual raises grave concerns about the psychological and physiological well-being of SCCY. Studies have shown that children who are sexually abused are more likely to engage in high sexual risky behaviors [27]. In addition, long term mental effects such as post-traumatic stress disorders as a result of these forms of abuse are common among SCCY [20]. There is therefore a need to develop interventions for both the victims and perpetrators of sexual abuse if we are to address this problem. This is because we hypothesis that the perpetrator of this form of abuse may have been or are victims themselves.

On the contrary, street boys were socialized to be aggressive and hostile, hence physical humiliation and bullying formed a great part of their induction. In addition, volatile substance use (primarily sniffing glue) and eating of garbage was encouraged. Even though sniffing of glue was not cited as an induction rite for girls it may have occurred. We propose that this may be a conscious effort by the older street members to prepare the new SCCY for the harsh reality of street life. Unfortunately, it presents additional health risks [16] including drug addiction [16–18] and mental health disorders [20].

A previous study in Kenya showed that SCCY used complex body language and appearance as a symbol of identity and cohesion [9]. In our study, smearing of the entire body with black soot formed the most defining symbol of acceptance. It was a form of identity used to distinguish street members from the rest of the society. We propose that it may be a unifying projection of their rebellious nature, given the hostility they receive from the larger community. Further studies are needed to better understand these rituals.

It was somewhat surprising that the street community was not easily accepting of any new boy or girl. Interrogations were done to screen out those who they believed deserved to live on the streets based on their home environments. This projects a more humane and protective side of the street community. It also suggests that they believed that the street was not an ideal environment for children who had no valid reason for being there. Unfortunately for SCCY girls, interrogation was a way for street boys to obtain new sexual partners. New SCCY girls are encouraged to accept sexual advances from any street boy as a form of protection from more adverse outcomes such as gang rape. These findings support the high rates of early sexual debut, pregnancy and sexual transmitted infections reported among this group [13–16, 19].

Street communities are governed by their own rules and regulations [9, 10]. It is possible that these rules may be grounded on past hostile and abusive experiences and further studies are need to better understand how these rules are established. With lack of parental or societal guidance as well as low education levels [12], also evident in our findings, what is viewed by the larger society as abusive, to the street community, is mundane. The fact that these children remain on the streets with hostile conditions signifies the sense of belonging, value, and solidarity they place in their structures [9]. It also highlights that to them being on the streets is much better than being back in their homes. Hence even though repatriation may be a noble idea, it may not often be feasible or appropriate due to the trauma and violence faced by SCCY. A non-judgmental approach that appreciates the structures and rules embedded within street communities may be critical to redefining the street culture and developing an appropriate repatriation model for SCCY. Health programs will need to fully engage SCCY [9] in developing these approaches.

Finally, our study highlights major gaps in the government and community structures for preventing and addressing physical and psychological abuse within the street community. The fact that there were no mechanisms to address sexual abuse among SCCY was concerning. SCCY perceived that law enforcers generally did not value the cases of abuse reported by street members, where the victims and perpetrators were SCCY. The government, law enforcers, and existing children rights organizations will need to critically think of appropriate ways to address violence and abuse prior to and during street induction. In addition, there is an urgent need for interventions to uphold and promote child protection rights in order to prevent vulnerable children from ending up in the streets, and once there, supporting them [28].

This study is not without limitations. The sampling procedure limits the generalizability of the study. Moreover, children who agreed to participate may have been systematically different from those who did not participate in the study or those whom we could not recruit (girls 11–13 years). Furthermore, social desirability bias could have influenced the way SCCY responded to sensitive questions regarding sexual practices. Lastly, the study findings are based on secondary data analysis following a primary study exploring sexual behaviors of SCCY. Hence, our findings are limited to the data obtained from the primary study. Further studies that apply sound theoretical approaches to better understand initiation practices including gang formation, street exit strategies, and long-term effect of these rituals on their overall wellbeing are needed. Despite these limitations, our study provides preliminary findings into the experiences of SCCY as they are initiated into street life. These findings have rich implications for interventions and policies to mitigate negative outcomes associated with street-involvement and promote the health of SCCY in Kenya.

## Conclusion

In conclusion, our study reveals that the street community has well established roles and rules that are sometimes protective of their members and viewed as critical for survival in the street. Unfortunately initiation rituals exact a heavy and destructive price, especially to girls. The rituals expose SCCY to adverse health risks including early pregnancies, STI, HIV, injuries and mental illness. Our findings provide valuable information about the health risks that SCCY are exposed to by their fellow SCCY as they are initiated into street life. These findings are important for the development of appropriate interventions for this vulnerable group.
